# Parasitism and Physiological Trade-Offs in Stressed Capybaras

**DOI:** 10.1371/journal.pone.0070382

**Published:** 2013-07-24

**Authors:** Ayelen T. Eberhardt, Sebastián A. Costa, M. Rocío Marini, Andrea Racca, Cecilia J. Baldi, M. Rosario Robles, Pablo G. Moreno, Pablo M. Beldomenico

**Affiliations:** 1 Laboratorio de Ecología de Enfermedades, Instituto de Ciencias Veterinarias del Litoral, Universidad Nacional del Litoral - Consejo de Investigaciones Científicas y Técnicas (UNL - CONICET). Esperanza, Santa Fe, Argentina; 2 Cátedra de Patología Básica, Facultad de Ciencias Veterinarias, Universidad Nacional del Litoral. Esperanza, Santa Fe, Argentina; 3 Centro de Estudios Parasitológicos y de Vectores (CONICET) La Plata, Buenos Aires, Argentina; Institut Pluridisciplinaire Hubert Curien, France

## Abstract

Parasites play a key role in regulating wildlife population dynamics, but their impact on the host appears to be context-dependent. Evidence indicates that a synergistic interaction between stress, host condition and parasites is implicated in this phenomenon, but more studies are needed to better understand this context-dependency. With the goal to assess the net effect of two types of chronic stress on various host-parasite interactions, we conducted an experiment in capybaras to evaluate the impact of food restriction and physical restraint on the infection intensity of specific gastrointestinal nematodes and coccidia, and how these stressors affected the growth, body condition, and some immuno-physiological parameters. Our hypothesis was that both forms of stress would result in an alteration in the host-parasite interactions, with deteriorated condition and reduced immunological investment leading to high parasite burdens and vice versa. Stressed capybaras had significantly higher coccidia infection intensities; but among individuals that were smaller, those stressed consistently showed lower helminth burdens than controls. Both stress treatments had a marked negative impact on growth and body condition, but concomitantly they had a significant positive effect on some components of the immune system. Our results suggest, on the one hand, that during prolonged periods of stress capybaras preventatively invest in some components of their immunity, such as innate humoural defenses and cells that combat helminths, which could be considered a stress-dependent prophylaxis. On the other hand, stress was found to cause greater infection intensities of protozoans but lower burdens of nematodes, indicating that the relationship between stress, physiological trade-offs and infection depends on the type of parasite in question. Moreover, both findings might be related in a causal way, as one of the immunological parameters enhanced in stressed capybaras is associated with the immune response to control helminths.

## Introduction

Mounting empirical evidence supports the notion that parasites play a key role in wildlife population dynamics [1]–[3]. Parasites are detrimental to the health of their hosts and can cause a decrease in their probability of survival and/or reproduction by exerting a specific pathogenic effect, extracting host’s resources and/or inducing a nutritionally demanding immune response [4]–[6]. Due to this, parasites have considerable potential to regulate the growth of host populations in nature [7], which has been experimentally demonstrated for some host-parasite systems [2], [3]. However, the impact of a parasite on its host appears to be context-dependent [8].

Although the factors that determine a context that enhances parasite virulence are yet to be elucidated, some hypotheses have been suggested. Lochmiller and Deerenberg posited that nutrient limitation is amongst the most important environmental stressors that influence immunocompetence and, subsequently, population regulation [5]. Some endemic parasites may be only rarely pathogenic, but they could become important population regulators when hosts are stressed [8]–[10]. A few studies show that certain components of the immune system are enhanced by stress [11]. However, it is widely accepted that prolonged (chronic) stress decreases immune function through mechanisms involving the hypothalamic-pituitary-adrenal (HPA) axis and glucocorticoids, leaving individuals more susceptible to infection [12], [13]. It has been suggested that this stress-dependent vulnerability of the host is the mechanism by which parasites exert a control on their host populations, as hosts tend to be stressed and in poor condition (thus becoming more vulnerable to their parasites) when their densities are high [14].

In a recent study, Pedersen and Greives [3] demonstrated that the interaction between food availability, stress and parasites could drive oscillations in wild mouse populations. Furthermore, their study revealed a synergy between nutrient availability and parasitic infection. Beldomenico and Begon [14] suggested that such synergy might be caused by reciprocal effects between parasites and physiological condition. Individuals in poor condition are more susceptible to infections, which further weakens their condition and predispose them to even more severe infections, and so on, creating a vicious circle in which infection intensity is both a cause and consequence of the immune condition of the host. However, empirical data from multiple host-parasite systems are needed to enhance our understanding of the mechanisms by which parasites regulate host populations.

The capybara, *Hydrochoerus hydrochaeris* Linnaeus (Rodentia: Caviidae), the largest living rodent on earth, is one of the most intensely utilized wildlife species in South America [15]. Capybaras are hosts to a very rich parasite community, including several specific helminths and protozoans that show high prevalence and ubiquity [16], [17]. The nematodes most frequently reported in capybaras are *Strongyloides chapini* Sandground (Rhabditoidea, Strongyloididea), *Hydrochoerisnema anomalobursata* Arantes & Artigas (Trichostrongyloidea, Viannaiidae), *Viannella hydrochoeri* Travassos (Trichostrongyloidea, Viannaiidae), *Trychostrongylus axei* Cobbold (Trichostrongyloidea, Trychostrongylidae), *Protozoophaga obesa* Diesing (Oxyuroidea, Oxyuridae), *Trichuris* sp. Roederer (Trichinelloidea, Trichuridae) and *Echinocholeus hydrochaeri* Travassos (Trichinelloidea, Trichinellidae). Among the protozoans, the most common coccidia are *Eimeira hydrochoeri* Carini, *E. trinidadensis* Casas, Duszynski and Zalles, *E. ichiloensis* Casas, Duszynski and Zalles, *E. boliviensis* Casas, Duszynski and Zalles and *E. araside* Gurgel, Sartori and Araújo [18]. Capybara population dynamics studies showed density-dependent effects on body mass gain, fecundity, survival of newborn and mortality of adults [19], but the involvement of parasites in these effects have not been investigated. Despite the large number of gastrointestinal parasites found in capybaras, no associated pathology has been described. Nevertheless, there are reports of negative associations between body condition and helminth intensity for *V. hydrochoeri* and the cestode *Monoecocestus macrobursatum* [20].

Previous studies have examined separately the impact of nutritional restriction and other forms of stress on growth [21], immunity [22], and parasites [9]. Here we assess these outcomes altogether to establish the net effect of two types of stress on host-parasite interactions, producing evidence that might shed some light on the underlying interactions and trade-offs. We evaluated the impact of food restriction and stress induced by capture and restraint (physical stress) on the parasitism intensity of specific gastrointestinal nematodes and coccidia, and measured how these stressors affected the growth, body condition, immunological investment and other physiological parameters of capybaras. Our hypothesis was that both forms of chronic stress alter host-parasite interactions, with deteriorated condition and reduced immunological investment leading to high parasite burdens and vice versa in a circular process, as in the vicious circle hypothesis mentioned above.

## Materials and Methods

### Ethics Statement

The animal care and treatments used in this experiment were approved by the Bioethical Committee of the School of Veterinary Medicine of Universidad Nacional del Litoral (Permit Number: 36/09).

### Animals and Enclosures

Capybaras are gregarious rodents in the suborder Histricognathi (cavy-like rodents) that inhabit South American wetlands. Adult weight, which is around 60 Kg of body mass, is reached after they are 2 years old, and their lifespan range from 10 to 14 years [23]. Females reach physiologic puberty between 12 and 18 months of age.

The experiment was carried out in specially designed enclosures built at the experimental zoological station “Granja la Esmeralda” in Santa Fe, Argentina. All enclosures were identical, measuring 7×3.5 m, with soil ground, each including a 1.75×1.50 m shelter, a tank for water, and half of its surface was covered by a cloth shade to provide protection from direct sunlight. The enclosures were built contiguously, creating a big rectangle, and a cloth shade covered the subdividing fences to prevent capybaras from seeing other enclosures (Figure S1 in supporting information). The capybaras that were used in the experiment were acquired from the commercial farm “Ayuí” (owned by heirs of Julio Cesar Storti), Santo Tomé, Corrientes, where they are bred and kept in semi-captivity until sold for their meat and pelt, and parasitism occurs naturally and is not controlled by antiparasitic drugs. To reduce the variability induced by sex and age on the studied variables, the individuals used in the study were all females between 6 and 12 months old at the beginning of the experiment. At the outset, the study began with 30 individuals (5 animals per enclosure), but soon three needed to be excluded due to different causes. One was mis-sexed, one died immediately after the translocation and another one escaped and could not be found for several days. The remaining 27 were allocated to the six enclosures through stratified random sampling to ensure an even distribution of initial body mass (which also determines that ages are distributed homogeneously, as body mass correlates very strongly with age during the first two years of age [24]). Half of the enclosures received five animals and the other half, four. Table S1 in supporting information shows a summary of the experimental design. Water was available *ad libitum* for the whole duration of the experiment. Veterinarians inspected the capybaras daily making sure that no clinical signs of disease were apparent. The only medical treatment that was administered during the experiment was the use of insecticide spray on a wound to prevent infestation by maggots (one individual, single application).

### Acclimation and Baseline Comparisons

Before beginning the treatments, the animals were left to acclimate in their new environment for four weeks, during which they were fed *ad libitum* and were not subject to capture and physical restraint. The meals were administered twice a day and consisted of fresh alfalfa, hay (sorghum or maize) and a mixture of rice bran and rice meal. The food was provided on the ground, one lump per individual to avoid differential access to food due to social hierarchy. During these weeks the total daily consumption per capybara was determined to be 800 gr. of mixture of rice bran+meal, 300 gr. of sorghum or maize hay, and 500 gr. of fresh alfalfa.

Also, this 4-week acclimating period was used to carry out baseline comparisons that assured that the treatment groups were not different at the beginning of the experiment in terms of body mass and size, body mass index, and faecal parasite egg and oocyst counts. These comparisons were made using Kruskal-Wallis tests. For these baseline comparisons only, *α* was set at 0.1 to reduce the probability of a type II error. A statistically significant test indicated that a re-allocation into enclosures via a new stratified random sampling was needed.

### Treatments

Three treatments were established: animals in two of the enclosures were fed a restricted diet (hereafter, food restricted group), others in two different enclosures were captured and physically restrained three times a week (physically stressed group), and the individuals in the remaining two enclosures served as control groups (Table S1). The treatments were spatially distributed in a way that ensured that enclosures with controls and food-restricted groups were adjacent to a physically stressed group (Figure S1).

The treatments were implemented for twelve consecutive weeks, and commenced immediately after the four acclimation weeks were completed. Each treatment was applied to 9 capybaras (in two enclosures). The food restricted group was provided with a diet of 50% less rice bran+meal (400 gr. per capybara) and of 40% less hay and fresh alfalfa (150 gr. and 300 gr., respectively) than that consumed when fed *ad libitum* during the acclimation period. Dietary restriction while avoiding malnutrition can be accomplished by a 20 to 60% reduction from average unrestricted food intake, including balanced decrease in calories, protein, vitamins, and minerals [25]. Three times a week (on Mondays, Wednesdays and Fridays), animals in the physically stressed group were chased, captured using a net and then physically restrained by tying their limbs for 10 minutes. They were fed 800 gr. of mixture of rice bran+meal, 300 gr. of sorghum or maize hay, and 500 gr. of fresh alfalfa. The control group was fed the same diet as the physically stressed groups but they were not stressed by capture and restraint.

After twelve weeks of treatment, all animals were anesthetized with 10 mg.kg^−1^ ketamine and 0.5 mg.kg^−1^ xylazine [26] and euthanized by exsanguination immediately after collecting a blood sample from the cava vein and recording the body mass and morphometric measures. Later, they were necropsied to obtain samples from different organs.

### Stress Assessment

In order to evaluate if the treatments were inducing measurable stress on the capybaras, we compared the proportion of the adrenal cortex that corresponded to the fascicular portion, which is the part of the gland that synthesizes glucocorticoids in response to stress [27]. The adrenal glands fixed in 10% buffered formalin were dehydrated in a graded series of ethanol, cleared in xylene and embedded in paraffin. Serial sections were cut at 5 µm, mounted on glass slides and stained with hematoxylin and eosin. Stained sections were examined under a light microscope at 200× and measured at 1000× using an eyepiece graticule.

The behaviour of the animals along the experiment indicated that habituation of individuals or stressing of the wrong group was not occurring. For the whole duration of the experiment, individuals from the physically stressed group acted with conspicuous evasive behaviour as soon as a person approached the enclosure, while this was not observed in capybaras of the other two treatments.

### Measures of Growth and Body Condition

Before and after the experiment, all individuals were weighed (initial body mass, *IBM*; final body mass, *FBM*) using a mechanic scale (precision 0.2 kg.). The morphometric measure used in this study was total length (*TL*). The measures of growth and body condition used were, body mass gain (*FBM - IBM*), body mass index (*FBM/TL*) [24], and body condition score. The latter was estimated by palpating the fat and muscle cover over the thoracic vertebrae and pelvic bones [28]. Each area was scored on a scale between 1 and 5 and both scores were summed. Body condition scoring was always carried out by the same person (ATE).

### Measures of the Immune System and Other Physiological Parameters

We evaluated the effect of the treatments on physiological parameters associated with the immune system and general health of mammals. Once animals were anesthetized, a 15–20 ml blood sample was taken from the cava vein and stored in tubes with and without anticoagulant (EDTA 10%). Samples with anticoagulant were kept refrigerated and processed within 8 h of collection. The other sample was centrifuged and the sera obtained were kept frozen at −20°C until further processing. At necropsy, the spleen was weighed using a digital scale and the adrenal glands were stored in 10% buffered formalin.

The sample with anticoagulant was used to produce blood cell counts. They provided an indication of aerobic capacity and energetic balance (red blood cells, RBC), and immunological investment (white blood cells, WBC) [29]. Whole blood was diluted 1∶10 in saline solution, and then used to produce two final dilutions: 1∶20 in 3% acetic acid and 0.5% of methylene blue (Türk solution) and 1∶200 in saline solution to count WBC and RBC, respectively, using Improved Neubauer’s chambers [26]. The remaining blood was used to produce blood smears for differential WBC counts to estimate the levels of Lymphocytes, Neutrophils, Monocytes, Eosinophils and Basophils. Smears were fixed and stained with May Grunwald-Giemsa. At least 200 WBCs were counted.

Serum samples were used for the determination of total plasma protein (TPP), albumin (A) and globulins (Gb) [30] using a colorimetric assay (Proti2, Wiener lab., Rosario, Argentina). The A/Gb ratio was also analysed. Variations in the A/Gb ratio may indicate underproduction (high ratio) or overproduction (low ratio) of antibodies, or underproduction of albumin (low ratio) [31].

Other measures of immunological investment used were the spleen mass index, calculated by dividing spleen mass by initial body mass (spleen mass/*IBM*) [9], [22] and natural antibodies (NAb) titers [32]. The determination of NAb titers was done by a hemagglutination assay described by Matson and collaborators [32], with some modifications. Briefly, serum samples (25 µl) were added to columns 1 and 2 of 96-well round bottom plates (Corning Costar) and 25 µl of PBS were added to columns 2 to 10, so that the second well contained a 1∶2 dilution. Doubling dilutions were made by transferring 25 µl to from one well to the next, up to well 10, leaving well 11 as positive control (IgM monoclonal antibody specific to α-galactosamine of glucoproteins and glucolipids; Alexis Biochemicals) and well 12 as negative control (PBS only). A stabilized rabbit red blood cells suspension (1.5%) was added to each well and the plates were mechanically shaken for several seconds followed by incubation for 2 h at room temperature. Titers were recorded as the column number of the last serum dilution showing clear evidence of agglutination.

### Parasites

We chose to study direct-cycle parasites because burdens of those with an indirect cycle (i.e. cestodes and digeneans) depend directly on the intake of intermediary hosts, which would be very difficult to recreate under our experimental conditions. At necropsy, the entire gastrointestinal tract was removed. The stomach, small and large intestines, and caecum were isolated by ligature. Each section was then opened and the contents washed into separate measuring containers. Mucous membranes were extensively washed and scraped under running water to remove adhered worms [33]. Due to the large volume of caecal and small intestine contents, we estimated helminth intensity at these sections by taking aliquots, which have shown to be good estimators of the entire gut section in other mammals [33]. The contents were put into measuring pails, stirred to homogenize, and a 10% aliquot was collected. The large intestine was opened and examined macroscopically during the necropsy. These aliquots were fixed in 5% buffered formalin and then examined under microscope to classify and count helminths. Species identification was done using specific keys and descriptions [34]–[47].

For baseline comparisons in the 4-week acclimation period, we used faecal egg/oocyst counts. Fresh faecal samples from each individual were evaluated for coccidian oocysts and helminth eggs using a modified Wisconsin Sugar Flotation quantitative method [48] at Laboratorio de Estudios Parasitológicos, Facultad de Ciencias Veterinarias, Universidad Nacional del Litoral. We also used this technique to estimate the coccidian infection intensity at the end of the experiment.

### Statistical Analysis

Given the nature of the treatments implemented, capybaras that received a given treatment needed to be confined in the same enclosure (e.g. chasing one capybara stresses out the whole lot in the enclosure). Although the enclosures were identical and contiguous, an individual receiving a given treatment shared the enclosure with other 3 or 4 individuals receiving the same treatment. Because observations from the same enclosure were not independent, an effect attributed to a treatment might in fact be an “enclosure” effect. To account for this lack of independence of observations from individuals in the same enclosure we used linear mixed models (LMM) or generalized linear mixed models (GLMM) with ‘enclosure ID’ as a random intercept, which takes into account that groups of observations belong to the same enclosure [49], [50]. The type of model used (LMM or GLMM) was decided on the basis of the distribution of the response. When the distribution of the response was approximately normally distributed or it could be transformed to approximate normality by exponentiation, a LMM was used. All parasite counts showed an aggregated distributional pattern, for which GLMM with a negative binomial response was the most appropriate model type [51].

To assess the impact of induced stress on body mass gain, body condition and immuno-physiological parameters, we used LMM, transforming by exponentiation the response variable to approach normality, where appropriate. We used the *lme4* [52] and *languageR* [53] packages of the statistical software R (functions *lmer* and *pvals.fnc*, respectively). The initial models had the main effects *Treatment* and *IBM,* and the interaction term Treatment*IBM, to take into account the potential effect modification of differential *IBM*, as well as its potential confounding effect. A likelihood ratio test was used to assess the significance of the interaction term. When the interaction term was not-significant, it was removed from the model and only the main effects were retained (whether significant or not).

To evaluate the impact of induced stress on the intensity of infection of selected parasite species, we used GLMM with negative binomial responses (*glmmADMB* [54] package of R). The initial models included the same main effects and interaction as the LMM above. Removal of unimportant interaction terms from the models was done by likelihood ratio test as described above. The data will be made freely available upon request.

## Results

The descriptive statistics of the parameters measured in the capybaras are shown in Table 1. Baseline comparisons carried out during the four weeks prior to establishing the treatments showed that experimental groups were not significantly different in terms of body mass and body mass index, and with respect to faecal parasite egg/oocyst counts of all parasite species investigated (*p*>0.1 for all comparisons).

**Table 1:** Parameters of health (including physiological values, immunological parameters, growth and body condition measures) of captive capybaras under three different feeding and physical stress regimes.

Health parameters	Control group		Food restricted group		Manipulation group	
	LI	HI	LI	HI	LI	HI
	Mean (Range)	Mean (Range)	Mean (Range)	Mean (Range)	Mean (Range)	Mean (Range)
Adrenal Fascicularproportion (%)	73.7 (64.0,86.7)	75.6 (72.7,77.4)	76.9 (68.9,82.3)	78.8 (77.4,81.2)	82.2 (78.2,85.7)	74.8 (66.7,81.1)
Body mass gain (kg)	4.2 (3.0,5.5)	4.4 (3.0,6.5)	1.0 (–0.5,3.0)	1.5 (1.5, 1.5)	3.6 (1.5,6.9)	1.2 (–1.0,4.0)
Body condition score	6.5 (6.0,7.0)	7.6 (7.0,8.0)	4.6 (3.0,5.5)	6.0 (6.0,6.0)	5.8 (5.5,6.5)	7.3 (6.0,8.0 )
Body mass index (bodymass/total length)	0.198 (0.19,0.21)	0.262 (0.23,0.30)	0.158 (0.10,0.18)	0.240 (0.22,0.27)	0.193 (0.14,0.25)	0.246 (0.22,0.29)
RBC (millons of cells/µl)	2.97 (2.06,3.75)	3.43 (2.30,5.05)	4.09 (3.00,4.95)	3.80 (3.27,4.34)	3.49 (3.41,3.64)	4.08 (3.25,5.30)
WBC (thousand of cells/µl)	6.58 (9.20,4.80)	6.58 (7.50,5.40)	6.37 (9.85,3.55)	8.57 (6.45,11.40)	6.57 (4.20,9.35)	6.59 (5.42,7.55)
L (thousand of cells/µl)	3.10 (1.84,4.44)	3.24 (2.51,4.29)	3.05 (2.16,4.44)	4.20 (3.20,5.03)	3.17 (1.99,3.93)	3.20 (2.33,4.28)
N (thousand of cells/µl)	2.97 (2.46,3.79)	2.75 (2.08,3.73)	2.25 (1.09,3.41)	3.50 (2.59,5.24)	2.59 (1.83,4.12)	2.62 (1.97,3.22)
E (cells/µl)	229 (170,391)	315 (70,716)	730 (121,2036)	702 (428,975)	580 (304,971)	477 (181,587)
B (cells/µl)	124 (0,222)	158 (0,422)	151 (0,415)	72 (0,185)	120 (60,185)	128 (26,219)
M (cells/µl)	126 (50,261)	108 (0,214)	168 (69,258)	83 (0,153)	101.23 (0,145)	166 (20,330)
Log2 NAb titer	7.0 (6,9)	7.8 (6,9)	8.8 (7,12)	11.3 (10,13)	6.7 (6,7)	5.8 (4,7)
Spleen mass index	3.61 (2.84,4.37)	3.07 (2.27,3.75)	2.99 (1.97,4.69)	2.81 (2.50,3.28)	3.23 (3.02,3.37)	2.74(2.22,3.81)
TPP (g/dl)	4.26 (3.14,5.26)	5.14 (4.54,6.22)	5.10 (4.46,5.59)	5.44 (3.90,7.02)	4.72 (4.09,5.59)	5.58 (4.85,7.01)
A (g/dl)	2.81 (1.64,3.60)	2.99 (2.80,3.60)	3.17 (2.58,3.58)	3.15 (2.88,3.42)	3.06 (2.69,3.28)	3.20 (2.52,3.84)
A/Gb (g/dl)	2.04 (1.10,3.18)	1.46 (1.02,1.84)	1.72 (1.04,2.36)	1.78 (0.81,2.82)	1.99 (1.35,2.73)	1.59 (0.56,2.18)

In this table, for reporting purposes, the median of initial body mass (17 kg) was used as the criterion to divide into body mass classes (lighter and heavier individuals).

Abbreviations: LI: lighter individuals at the beginning of the experiment; HI: heavier individuals at the beginning of the experiment; RBC: red blood cells; WBC: white blood cell; L:Lymphocytes; N: Neutrophils; E: Eosinophils; B:Basophils; M: Monocytes; NAb: natural antibodies; PTT: total proteins; A: albumin; Gb: globulins.

### Stress Assessment

An analysis of the histo-architecture of the adrenal cortex after the finalization of the experiment revealed that individuals belonging to both treatments had significantly greater fascicular portions than controls, indicating that treatments induced measurable stress on the capybaras (Table 2). The difference between controls and physically stressed individuals was greater among individuals that were initially lighter than among heavier individuals. This trend was also observed in food-restricted individuals, but the interaction term was not quite significant (p = 0.089) (Table 2).

**Table 2:** Linear mixed model describing the effect of treatments on the fascicular portion of the adrenal gland of capybaras (*N = *27).

Response = fascicular proportion (µm)				
Term	Coefficients	Standard error	*P*-value^b^	F-value^c^
Intercept	0.582	0.062	<0.001	
Treat. _(Food restricted)_ a	0.175	0.073	0.026	2.74
Treat. _(Physically stressed)_ a	0.327	0.076	<0.001	2.74
IBM	0.008	0.003	0.019	0.73
IBM: Treat. _(Food restricted)_	−0.007	0.004	0.089	8.33
IBM: Treat. _(Physically stressed)_	−0.015	0.004	0.001	8.33

a Simple contrasts – reference level: control (the coefficients reflect comparison with control groups).

b P-values obtained from Markov chain Monte Carlo samples (*pvals.fnc* function in R).

c For the factor ‘Treatment’: numerator degrees of freedom = 2; denominator degrees of freedom = 24.

### Measures of Body Mass Gain and Body Condition

**Figure 1:**
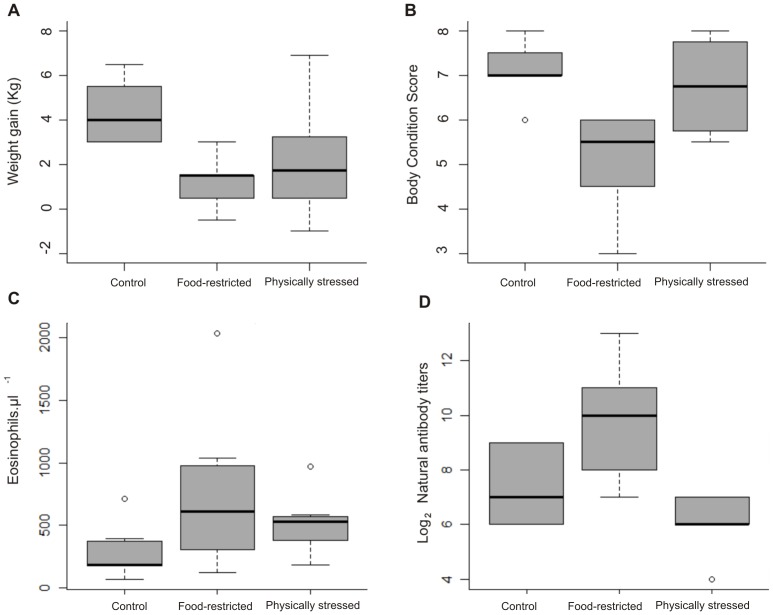
The effect of treatments on body mass gain, body condition score and immunological parameters. Boxplots showing the effect of three different feeding and physical stress regimes on, (A) body mass gain during the duration of the experiment; (B) body condition score; (C) eosinophil concentration in blood; and (D) natural antibodies titers. Boxplots depict the median (bold bar), 25–75% quartiles (box), 10–90% quantiles (whiskers) and outliers (points).

The effect of both treatments was largely evident on body mass gain, and it was also significant on body condition score and body mass index, although for these two the effect was only strong for food restricted individuals (Table 3; Figure 1). While control individuals gained on average 4.3 kg during the experiment, food restricted and physically stressed individuals grew only 25% (*p* = 0.0005) and 50% (*p* = 0.0178) that value, respectively.

**Table 3:** Linear mixed models describing the effect of treatments on body mass gain, body condition score and body mass index (*N* = 27).

Response = body mass gain				
Term	Coefficients	Standard error	P-value^b^	F-value^c^
Intercept	4.333	0.564	<0.001	
Treat. _(Food restricted)_ a	−3.294	0.798	<0.001	8.29
Treat. _(Physically stressed)_ a	−2.119	0.823	0.017	8.29
IBM	−0.060	0.058	0.314	1.06
**Response = body condition score**				
Intercept	4.803	0.396	<0.001	
Treat. _(Food restricted)_ a	−1.785	0.267	<0.001	34.24
Treat. _(Physically stressed)_ a	−0.578	0.274	0.0467	34.24
IBM	0.128	0.019	<0.001	43.73
**Response = body mass index**				
Intercept	0.109	0.012	*<*0.001	
Treat. _(Food restricted)_ a	−0.032	0.008	*<*0.001	19.98
Treat. _(Physically stressed)_ a	−0.018	0.008	0.040	19.98
IBM	0.007	<0.001	*<*0.001	138.72

a Simple contrasts – reference level: control (the coefficients reflect comparison with control groups).

b P-values obtained from Markov chain Monte Carlo samples (*pvals.fnc* function in R).

c For the factor ‘Treatment’: numerator degrees of freedom = 2; denominator degrees of freedom = 24.

### Effects of Treatments on the Immune System and Other Physiological Parameters

For blood cells, no differences between treatments were observed for red blood cells, lymphocytes, neutrophils and monocytes (Table S2). The only significant differences between treatments were found for eosinophils (Figure 1, Table 4). Individuals in the food restricted and physically stressed groups had eosinophil counts significantly higher than control individuals (*p* = 0.0113 and 0.0494, respectively) (Figure 1, Table 4).

**Table 4:** Linear mixed models describing the effect of treatments on eosinophils and natural antibody titers (*N* = 27).

Term	Coefficients	Standard error	P-value^b^	F-value^c^
Intercept	1.650	0.042	<0.001	
Treat. _(Food restricted)_ a	0.165	0.059	0.011	3.915
Treat. _(Physically stressed)_ a	0.122	0.061	0.049	3.915
IBM	0.004	0.004	0.348	0.917
Response = log2(NAb)^0.01^				
Intercept	1.020	0.001	<0.001	
Treat. _(Food restricted)_ a	0.003	0.001	0.011	10.84
Treat. _(Physically stressed)_ a	−0.002	0.001	0.051	10.84
IBM	0.000	<0.001	0.498	0.47

a Simple contrasts – reference level: control (the coefficients reflect comparison with control groups).

b P-values obtained from Markov chain Monte Carlo samples (*pvals.fnc* function in R).

c For the factor ‘Treatment’: numerator degrees of freedom = 2; denominator degrees of freedom = 24.

Individuals from the food-restriction groups showed significantly higher NAb titers than controls and physically stressed animals (*p* = 0.011 and *p = *0.002, respectively; Table 4; Figure 1). Physically stressed individuals showed a nearly significant (p = 0.051) trend to have lower NAb titers than controls.

Neither food restriction nor physical stress had a significant effect on TPP, A, Gb, A/Gb ratio or spleen mass (Table S2). However, there was an almost significant trend of food restricted individuals to have lower spleen mass than controls (p = 0.053).

### Impact of Induced Stress on the Intensity of Infection of Selected Parasite Species

**Figure 2:**
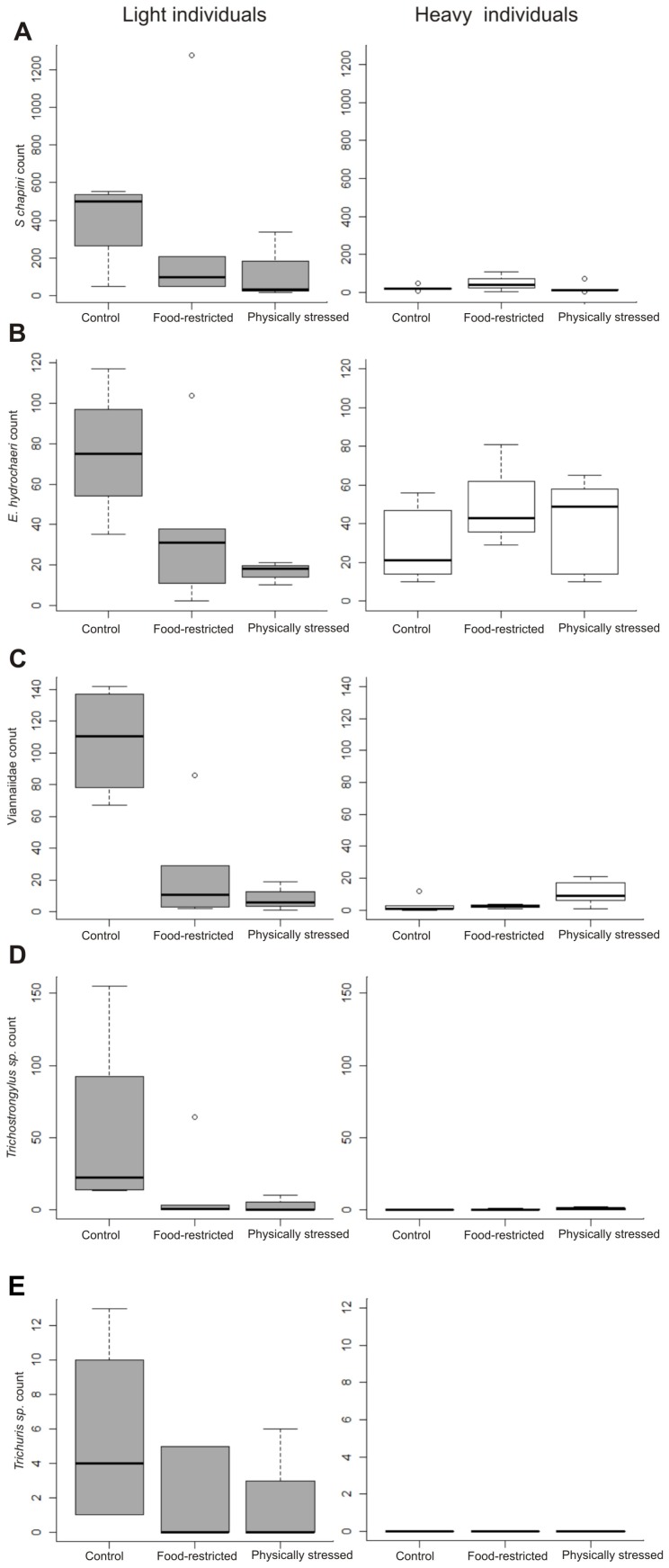
Intensity of different parasite species by treatments. In this boxplot, the median of initial body mass (17 kg) was used as the criterion to divide into body mass classes (lighter and heavier individuals). (A) *Strongyloides chapini*; (B) *Echinocholeus hydrochaeri*; (C) Family Viannaidae; (D) *Trichostrongylus* sp.; and (E) *Trichuris* sp. Boxplots depict the median (bold bar), 25–75% quartiles (box), 10–90% quantiles (whiskers) and outliers (points).

The nematodes found in necropsied capybaras were *S. chapini*, *Trichostrongylus* sp., *Trichuris* sp., *E. hydrochaeri*, *H. anomalobursata* and *V. hydrochoeri* (the latter two were counted together as ‘Viannaiidae’, because both females are undistinguishable). Although low counts of *P. obesa* eggs were occasionally observed at the beginning of the experiment, adults were not present at necropsy. The effects of treatments on nematode intensity depended on the capybara body mass at the beginning of the experiment for all nematode species found, except for *Trichuris* sp. (Table 5; Figure 2).

**Table 5:** Generalized linear model with a negative binomial response describing the effect of treatments on nematode and coccidian intensity (N = 27).

Response = *S. chapini*				
Term	Coefficients	Standard error	P-value	Deviance/Residual dev.
Intercept	10.288	1.188	<0.001	
Treat. _(Food restricted)_ a	−3.466	1.429	0.015	7.62/60.92
Treat. _(Physically stressed)_ a	−4.200	1.513	0.005	7.62/60.92
IBM	−0.325	0.062	<0.001	24.62/36.29
IBM*Treat. _(Food restricted)_	0.214	0.075	0.004	7.41/28.89
IBM*Treat _(Physically stressed)_	0.189	0.074	0.011	7.41/28.89
**Response = ** ***E. hydrochaeris***				
Intercept	5.249	0.875	<0.001	
Treat. _(Food restricted)_ a	−2.087	1.061	0.049	2.24/34.91
Treat. _(Physically stressed)_ a	−3.155	1.138	0.005	2.24/34.91
IBM	−0.078	0.047	0.099	1.12/33.79
IBM*Treat. _(Food restricted)_	0.111	0.058	0.058	5.81/27.99
IBM* Treat _(Physically stressed)_	0.140	0.058	0.016	5.81/27.99
**Response = ** ***Trichostrongylus*** ** sp.**				
Intercept	18.066	5.606	0.001	
Treat. _(Food restricted)_ a	−11.607	6.007	0.053	8.95/44.91
Treat. _(Physically stressed)_ a	−16.538	5.811	0.004	8.95/44.91
IBM	−1.057	0.383	0.006	9.60/35.31
IBM*Treat. _(Food restricted)_	0.674	0.412	0.102	15.05/20.26
IBM* Treat _(Physically stressed)_	0.993	0.389	0.010	15.05/20.26
**Response = Viannaiidae**				
Intercept	8.722	1.439	<0.001	
Treat. _(Food restricted)_ a	−3.558	1.739	0.040	10.59/45.68
Treat. _(Physically stressed)_ a	−6.707	1.774	<0.001	10.59/45.68
IBM	−0.311	0.074	<0.001	4.60/41.07
IBM* Treat. _(Food restricted)_	0.139	0.101	0.166	11.98/29.09
IBM* Treat _(Physically stressed)_	0.325	0.095	<0.001	11.98/29.09
**Response = ** ***Trichuris*** ** sp.**				
Intercept	10.452	3.012	<0.001	
Treat. _(Food restricted)_ a	−2.998	1.045	0.004	3.99/50.28
Treat. _(Physically stressed)_ a	−3.021	1.378	0.028	3.99/50.28
IBM	−0.648	0.209	0.001	39.91/10.37
**Response = Coccidian**				
Intercept	4.908	0.761	<0.001	
Treat. _(Food restricted)_ a	2.646	0.494	<0.001	26.27/33.40
Treat. _(Physically stressed)_ a	1.565	0.511	0.002	26.27/33.40
IBM	−0.074	0.038	0.051	3.42/29.97

a Simple contrasts – reference level: control (the coefficients reflect comparison with control groups).

When considering the initial body mass, light individuals in control groups at the end of the experiment had significantly greater *S. chapini*, *E. hydrochaeri*, Viannaiidae and *Trychostrongylus* sp. burdens than physically stressed groups (Table 5), while no significant difference was observed within heavier animals (Table 5; Figure 2). Food restricted capybaras showed the same trend (Figure 2), but the interaction was only statistically significant for *S. chapini* (Table 5).

In the case of *Trichuris* sp., both treatments showed lower parasite counts than controls (Figure 2, Table 5). The magnitude of the difference was much greater between controls and food restricted animals.

**Figure 3:**
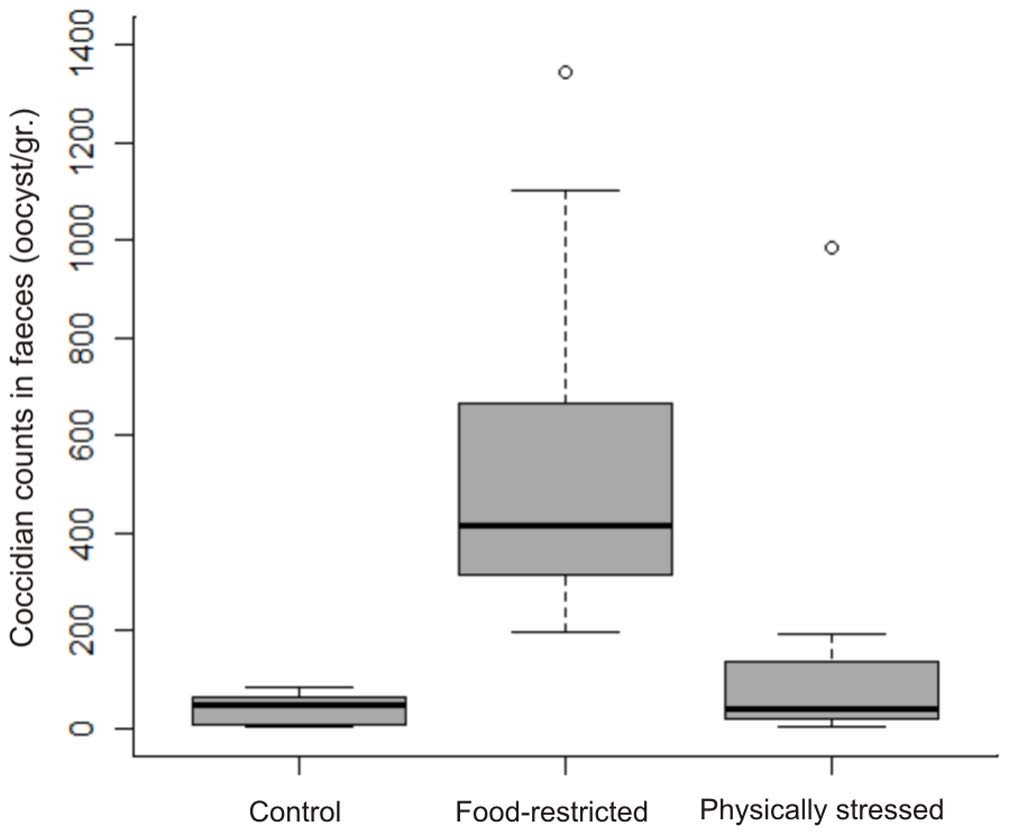
Coccidian infection intensity in capybaras under different treatments. Boxplots showing coccidian infection intensity (faecal oocyst count) at the end of the experiment in capybaras under three different feeding and physical stress regimes. Boxplots depict the median (bold bar), 25–75% quartiles (box), 10–90% quantiles (whiskers) and outliers (points).

Regarding coccidians, oocyst counts were much higher in both treatments than in control groups (Table 5). The magnitude and significance of this difference was much greater between controls and food restricted individuals (Figure 3). Food restricted individuals had significantly higher oocyst counts than physically stressed ones (*p* = 0.035).

## Discussion

The most ecologically significant results of this experiment were, on the one hand, that stress had a marked negative impact on the body mass gain and body condition of capybaras, but a positive effect on nonspecific components of the immune system: eosinophil counts were greater in capybaras from both treatment groups and NAb titers were greater in food restricted animals, compared to controls. On the other hand, the consequence of both treatments on the gastrointestinal parasitism dynamics depended on the type of parasite and on the size of the individuals at the beginning of the experiment. Stressed capybaras had significantly higher coccidian infection intensities than controls (this difference was much stronger in the food restricted group), but controls had, in general, higher helminth intensity than treated individuals among those that were lighter, and therefore younger, at the beginning of the experiment (especially in physically stressed capybaras). Our prediction that stressed capybaras would be in poorer condition, immunosuppressed and with high parasite burdens at the end of the experiment was only partially supported. Only for coccidian infection did we observe our expected scenario, although we did not find a significant decrease in any of the immunological parameters studied.

### Stress, Body Mass Gain and Body Condition

Large context-dependent differences in growth and body condition have been observed in some wild rodent populations. Voles and lemmings, for example, are larger (20–30% heavier) during the increase and peak phases of the population cycle than in the declining and low phases of their multi-annual fluctuations [55]. This phenomenon, referred to as the ‘Chitty’ effect, is believed to be a consequence of a dynamic allocation of energy among physiological functions [56]. In good years, rodents have a surplus energy that will allow continuous growth and deposition of additional body mass, while when the context is not favourable they suppress growth. Here we experimentally reproduced this context-dependency of somatic effort, showing that body mass gain and body condition are very sensitive to both nutritional and physical stress in capybaras.

Previous reports found that, under nutritional stress, rats and chicks reared under germ-free conditions had greater rate of growth than conventionally reared animals [21], [57]. This suggests a trade-off between growth and immunity, with immunity having priority over body growth when nutrients are limited [5]. The results of the present study are in agreement with the above, as animals under nutritional stress showed different priorities in their use of resources than non-stressed individuals, reducing their investment in somatic effort while enhancing some compartments of the immune system.

### Stress and Immunological Investment: Stress-induced Prophylaxis

The physiological parameters measured did not show the expected response to treatments. There was only a nearly significant trend of food restricted animals to have lower spleen mass index than controls (25% reduction in the spleen mass). A relationship between food restricted animals and reduced spleen size was found in an experiment with chicken [22]. It might be that this effect was not strong enough to be detected by the limited statistical power of our experiment.

Of the parameters measured, one showed to be sensitive to the influence of both types of stress, and a second one to nutritional stress, but in the opposite direction than predicted. While stressed individuals were significantly reducing their body mass gain and body condition, they were diverting part of their limited available energy to some nonspecific compartments of the immune system, as they had greater eosinophil counts and much higher Nab titers (the latter only in food restricted capybaras) than controls.

NAb are unique among the immunoglobulins, as their production does not require previous exposure to antigen [58]. Levels of NAb have been demonstrated in animals bred in pathogen-free environments [59]. Given that they confer nonspecific humoural immunity independent of pathogen exposure, NAb have the potential to be good indicators of the humoural immunocompetence of wild animals [60]. While it is known that chronic stress determines a reduction in specific antibody levels [61], how it affects Nab has been completely ignored. Our results suggest that nutritional stress enhances their production. Eosinophils, on the other hand, are key protagonists in the helminth-induced immune response, known as the T helper 2 cell (Th2) response, being the main leukocyte that increases their levels in the presence of helminth infections [62]. Contrary to our finding, chronic stress is known to cause a reduction in circulating eosinophil levels, as glucocorticoids suppress eosinophil proliferation and diminishes its survival [62].

This previous knowledge on NAb and eosinophils led us to anticipate that their levels would be lower in treated groups, as a result of immunosuppression caused by chronic stress and lack of resources to invest in immunity [12]. It is sensible to expect that individuals fed *ad libitum* and undisturbed should be able to invest more in immune function than stressed individuals. In this experiment, however, the only indication that stressed individuals were investing less in immune function was a not quite statistically significant reduction in the spleen size (25%). Moreover, investment in eosinophils was greater in stressed individuals, and NAb titers were much higher in food restricted animals. It is noteworthy that both TPP and the A/Gb ratio were not different between treatment groups (Table S2), indicating that it was unlikely that immunoglobulins other than NAb were also elevated in food restricted animals. Similarly, leukocytes other than eosinophils were not affected by treatments.

The stress-immunity interaction depends on coping styles (the behavioural and physiological efforts to master the stressing situation) [63]. Prey species often show a passive coping style, which is associated with high HPA reactivity and a Th2 biased immune response. The latter is because prey species are more likely to be exposed to macroparasites, as they show exploratory nature and greater intake of novel resources [64]. Increased eosinophils in stressed capybaras are reflecting an enhanced Th2 immune response in these individuals. It is unlikely that this difference in eosinophil counts was caused by differential helminth parasitism, as treated individuals had lower or similar nematode burdens as controls (faecal egg counts throughout the duration of the experiment were never higher in the treated groups; data not shown). This anti-helminthic immune response in the absence of increased helminth infection might be indicating preparedness in anticipation of greater risk of parasite exposure.

In rodents, the effect of stressors on the immune function depends on their duration [11]. They enhance immunity in the short term (hours-days) and cause immunosuppression when it is sustained for longer (weeks-months) [11]. Our results suggest that in capybaras chronic stress produces a diversion of resources to some nonspecific compartments of the immune system. Similar results were obtained by Hangalapura and collaborators [22] in chicken, who conducted an experiment and concluded that sustained food restriction did not have a significant effect on specific antibody responses, but rather suppressed parts of cell-mediated immunity and enhanced others of the innate immunity (increased production of reactive oxygen intermediates by phagocytes in whole blood). This suggests that the organism stressed over long periods of time invests in nonspecific immunity rather than in acquired cell mediated or humoural specific immunity, which makes biological sense. Firstly, because it makes little logic that organisms under chronic stress would suppress the whole immune system, as it could be critical for recovery from stressors [65]. Secondly, considering the wide diversity of parasites that infect a host under natural conditions, an optimal strategy may be to invest preemptively in defences against an array of pathogens rather than invest in a specific immune response to antigens of every member of the parasite community. This could be interpreted as a sort of stress*-*induced prophylaxis. A similar preventative strategy is used by some insects. When some lepidopteran species are reared under crowded conditions, they prepare for higher pathogen circulation by enhancing their immune system, resulting in butterflies that are significantly more resistant to infections than those reared solitarily, a phenomenon termed ‘density-dependent prophylaxis’ [66].

This stress-dependent nonspecific immune enhancement might explain some contradictory results observed in eco-immunological studies. For example, Vinkler and collaborators [67] found a negative association between the response to the phytohaemagglutinin skin-swelling test and ornament saturation of rosefinch males, indicating stronger immune responsiveness in inferior males. In that study, a major involvement of basophils in the swelling response was shown, which indicates the nonspecific nature of the reaction. The authors concluded that poor ornamentation was associated with greater immune response because both were indicative of low quality, as the immune response could be detrimental to the birds (inflammation is a destructive process). However an alternative interpretation of their findings is that, like in our experiment, stressed individuals invested more in nonspecific immunity at the expense of other physiological functions such as ornamentation. In such case, it would not be a matter of quality (genotypes) but rather of differential life-histories (phenotypes).

The physiological trade-offs observed in our experiment arise under stressing circumstances, thus allowing animals to adjust to changing environmental conditions. In favourable contexts, resources are readily available and the organism should be able to maintain its core body temperature, fight an infection, and produce and rear viable offspring concomitantly [68]. However, if resources are limited, the same organism will face severe challenges and will need to establish priorities in order to overcome the difficult moment. Its success will depend on the degree of stress faced by the animal. For example, an experimental study on tree lizards [69] showed that when food was unlimited, females were able to invest both in reproduction and heal their wounds. When food was restricted, they could still heal a cutaneous wound, but they needed to decrease reproductive investment, and under extreme food limitation both reproductive investment and wound healing were suppressed. We did not investigate different levels of stress in our experiment. It is to be expected, however, that capybaras would be unable to invest on eosinophils or NAb, should they be subject to a more severe stress.

Of course the differential costs of the various immune responses (Th1 v. Th2, specific v. generic, etc.) should be taken into account carefully in order to comprehend the trade-offs underlying changes in host-parasite interactions during stress. Further studies should focus on determining if the influence of stress on a specific compartment of the immune system depends on its relative cost compared to other compartments (e.g. are inexpensive immune responses enhanced and costly ones suppressed?).

### Stress and Parasite Intensity

Infection with species of *Eimeria* causes coccidiosis, a common disease of domestic animals. It is characterised by catarrhal to haemorrhagic enteritis that results in diarrhoea, anemia, dehydration, anorexia, weight loss and eventually death [70], [71]. We did not observe these signs, but as expected, both food restricted and physically stressed treatments had significantly greater oocyst counts than control groups, which could result in subclinical effects, as the severity of the enteritis is positively correlated with the infection intensity [72]. This result is in accordance with the notion of a vicious circle, as stressed individuals were in poorer body condition, and were also suffering more severe coccidian infections, which in turn would lead to an even more deteriorated condition [14]. However, no associated significant decrease in immune function was observed.

Although we expected similar results in the infection intensity of gastrointestinal nematodes, we observed the opposite. In individuals that were lighter at the beginning of the experiment, the pattern was consistent for all parasite species, controls had much higher parasite burdens than physically restrained individuals. The pattern held for food-restricted capybaras, although the difference was significant only for *Trichuris* sp. and *S. chapini.* We offer two not mutually exclusive hypotheses to explain this observation, and argue that the evidence gathered supports one over the other.

The first explanation relates to differential consumption rates of food resources. Unlike coccidians, which can multiply within the hosts like all microparasites, the intensity of nematode infection is strictly dependent on the amount of immature stages (eggs) that are ingested by a host. Individuals that were fed *ad libitum* would have greater intake of parasite eggs than those that had a lower consumption rate (food restricted). In addition, decreased host nutrition may negatively impact parasite populations through a simple reduction in resource availability [73]. However, the fact that the pattern of reduced helminth burdens in smaller capybaras was stronger or as strong in physically stressed capybaras than in food restricted ones argues against this hypothesis.

The other explanation would be that higher nematode intensity in controls might arise from a reduced parasite resistance (the ability to limit parasite burden) or increased tolerance (the ability to limit harm caused by a given burden). The lower levels of eosinophils in control individuals is an indication that they had less allocation of resources to immune response against macroparasites than stressed ones, which in turn could also lower parasite resistance [65], [30]. The measure of tolerance is the slope of the regression of host fitness on parasite burden [30]. In a supplementary analysis, we observed that the slopes of the relationship between body condition and parasite intensity were similar in all treatment groups (Table S3). This lack of significant differences in slopes suggests that tolerance was not affected by the treatments, but the difference in eosinophil counts indicates that resistance could have been affected. Therefore, this lower investment in Th2 response in control individuals was very likely the cause of the higher helminth infection intensity they suffered. What remains to be explained is why this was mainly observed in individuals that were smaller at the beginning of the experiment. This could be related to the immunological experience (acquired immunity), which would be greater in older individuals, but it could also be attributed to differential effect of stress on smaller and larger individuals. The latter was reflected in the adrenal glands, as the signs of stress were greater among physically restrained individuals that were initially lighter than among heavier individuals. Food restricted showed a similar trend, although the interaction term was not significant. In addition, even though all capybaras in our experiment were still growing, younger animals (i.e. initially lighter) were at a more demanding phase of body growth than older individuals [24], which constitutes an additional source of stress.

Further studies should explore the involvement of stress on the interaction between the immune response to typical intracellular parasites (e.g. coccidians) mediated by Th1, and that against extracellular parasites (e.g. helminths) primarily mediated by Th2, as cytokines produced by Th1 cells may suppress Th2 immune function and vice versa [74]. The immune response of capybaras might be Th2 biased, as explained above for passive coping species [63], which is in agreement with our findings; stress resulted in higher infection intensities in a parasite that elicits a Th1 response, while the infection intensities of those that are controlled by a Th2 response were indeed lower. It would be important to know what consequences in the life-history of capybaras result from these changes in the susceptibility to different parasite types induced by stress. Our results suggest that during stressful periods capybaras might be more vulnerable (although not necessarily more exposed) to microparasites like coccidians than to macroparasites.

### Conclusions

Although short to medium duration stressors elicit an enhancement of immunity, it is widely accepted that chronic stress results in immunosuppression through neuroendocrine mechanisms involving the HPA axis and glucocorticoids, in order to redirect resources towards physiological processes that are important to overcome the adverse situation [11], [13]. Although we have only assessed a limited set of components of the complex vertebrate immune system, we were able to show that this chronic stress-mediated immunosuppression does not hold for the whole immune system in capybaras. Our results suggest that parts of the nonspecific arm of the immune system are favored over specific components during stress, preparing the organism to face its very diverse parasite community with generic defences instead of investing in specific immunity to every single parasite species and opportunistic infectious agents they are exposed to. Animals use many environmental signals to modify their phenotypes in preparation for recurrent environmental challenges [11]. We named the immunological preparedness observed here ‘stress-dependent prophylaxis’.

We also showed that food restriction and physical stress had opposite influences on gastrointestinal microparasites and macroparasites. This highlights that special attention should be placed on the parasite type or species involved when attempting to interpret how the synergistic interaction between food availability, stress and parasites affects individual fitness and population dynamics.

## Supporting Information

Figure S1 **Schematic representation of experimental enclosures.** All enclosures were identical, measuring 7×3.5 m, with soil ground, each including a 1.75 × 1.50 m shelter, a tank for water, and half of its surface was covered by a cloth shade to provide protection from direct sunlight. The treatments were spatially distributed in a way that ensured that enclosures with controls and food-restricted groups were adjacent to a physically stressed group.
(TIF)

Table S1 **Summary of the design used for the capybara experiment.** Treatments were applied for 12 consecutive weeks following 4 weeks of acclimation.
(DOCX)

Table S2 **Linear mixed models describing the effect of treatments on red blood cells, white blood cells (lymphocytes, neutrophils, monocytes), neutrophil:lymphocyte ratio, spleen mass, plasmatic proteins, albumin, globulins and albumin:globulin ratio (** ***N*** **= 27).**
(DOCX)

Table S3 **Linear models showing the relationship between parasite load and body condition.** The inclusion of the interaction term Treatment*Parasite allowed assessing whether there was a change in the tolerance to parasites induced by stress. Body condition was negatively associated with *Trichuris* sp. and *Strongyloides chapini*. In no case, the interaction term Treatment*Parasite was significant, indicating no influence of treatments on parasite tolerance.
(DOC)
