# Supplementary figures and images for: Parasitism and Physiological Trade-Offs in Stressed Capybaras
Source: PLoS One. 2013 Jul 24;8(7):e70382. doi: 10.1371/journal.pone.0070382 (PMC3722164; doi:10.1371/journal.pone.0070382)

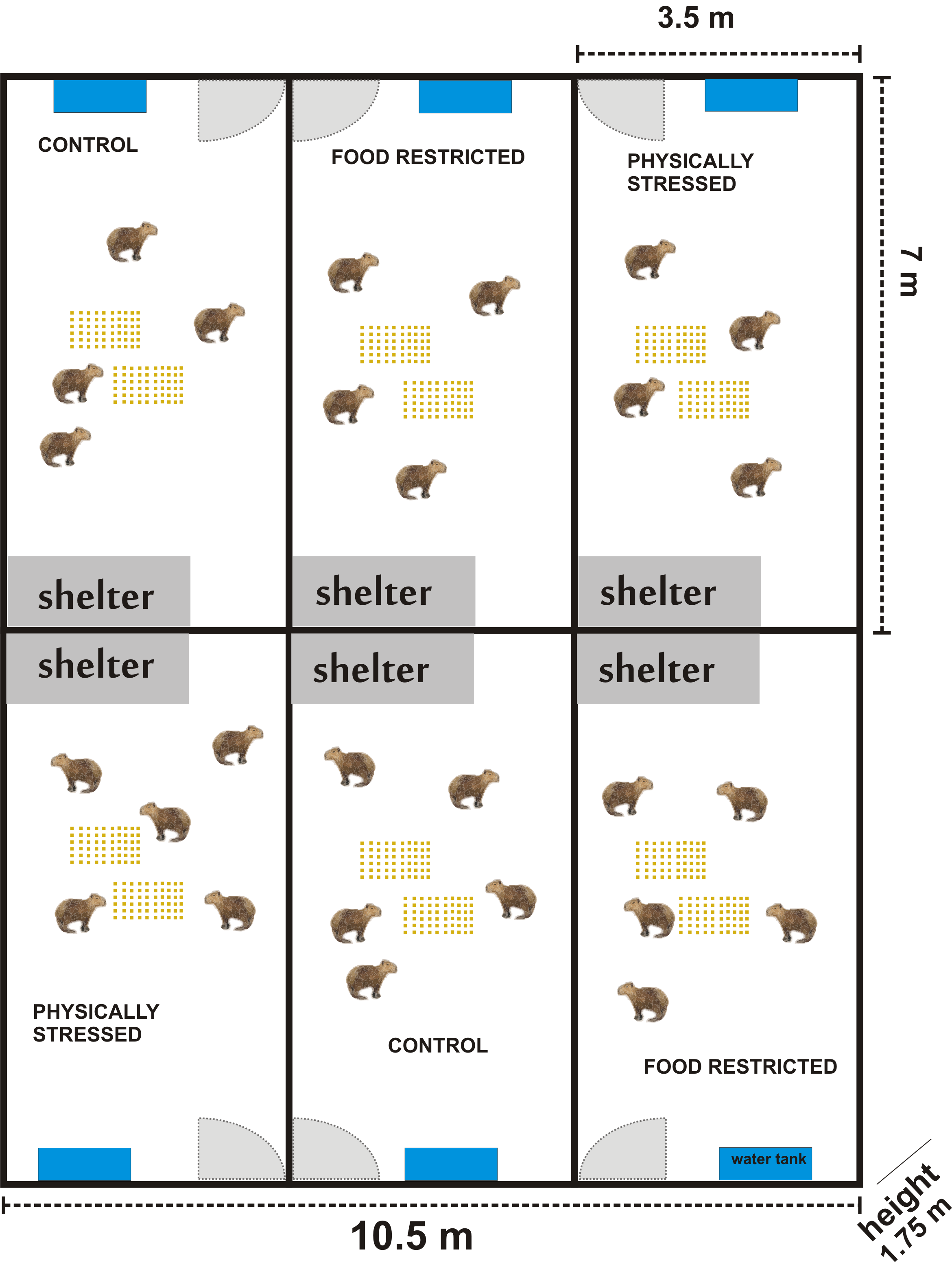

Supplement: Figure S1 — Schematic representation of experimental enclosures. All enclosures were identical, measuring 7×3.5 m, with soil ground, each including a 1.75 × 1.50 m shelter, a tank for water, and half of its surface was covered by a cloth shade to provide protection from direct sunlight. The treatments were spatially distributed in a way that ensured that enclosures with controls and food-restricted groups were adjacent to a physically stressed group. (TIF) [file pone.0070382.s001.tif]
