# Supplementary material for: Parasitism and Physiological Trade-Offs in Stressed Capybaras
Source: PLoS One. 2013 Jul 24;8(7):e70382. doi: 10.1371/journal.pone.0070382 (PMC3722164; doi:10.1371/journal.pone.0070382)
Supplement: Table S1 — Summary of the design used for the capybara experiment. Treatments were applied for 12 consecutive weeks following 4 weeks of acclimation. (DOCX) [file pone.0070382.s002.docx]

Table S1: **Summary of the design used in the capybara experiment.** Treatments were applied for 12 consecutive weeks following 4 weeks of acclimation.

| **Group** | Food restricted | | Physically stressed | | Control | |
| --- | --- | --- | --- | --- | --- | --- |
| Enclosure ID | A | B | C | D | E | F |
| Number of capybaras | 5 | 4 | 5 | 4 | 5 | 4 |
| Capybara/m^2^ | 0.20 | 0.16 | 0.20 | 0.16 | 0.20 | 0.16 |
| Capture and 10' restrain | Never | | Three times per week | | Never | |
| Food | 400g rice bran  150g hay  300g fresh alfalfa | | 800g rice bran  300g hay  500g fresh alfalfa | | | |
| Water | Ad libitum | | | | | |
| Baseline comparisons | Body mass and size; body mass index; faecal parasite egg and oocyst counts | | | | | |
| Measures of stress | Proportion of the adrenal cortex that corresponds to the fasciluar portion | | | | | |
| Measures of growth and body condition | Body mass gain; body mass index; body condition score | | | | | |
| Measures of the immune system | Total white bood cell counts; lymphocytes; neutrophils; monocytes; eosinophils; natural antibodies; spleen mass index | | | | | |
| Other physiological parametres | Total plasma protein; albumin; globulin; albumin/globulin ratio; red blood cells | | | | | |
| Parasites | Adult count for nematodes; faecal oocyst count for coccidians | | | | | |
