# Supplementary material for: Parasitism and Physiological Trade-Offs in Stressed Capybaras
Source: PLoS One. 2013 Jul 24;8(7):e70382. doi: 10.1371/journal.pone.0070382 (PMC3722164; doi:10.1371/journal.pone.0070382)
Supplement: Table S2 — Linear mixed models describing the effect of treatments on red blood cells, white blood cells (lymphocytes, neutrophils, monocytes), neutrophil:lymphocyte ratio, spleen mass, plasmatic proteins, albumin, globulins and albumin:globulin ratio ( N = 27). (DOCX) [file pone.0070382.s003.docx]

Table S2. Linear mixed models describing the effect of treatments on red blood cells, white blood cells (lymphocytes, neutrophils, monocytes), neutrophil:lymphocyte ratio, spleen mass, plasmatic proteins, albumin, globulins and albumin:globulin ratio (*N*= 27).

| Response= Red blood cells | | | | |
| --- | --- | --- | --- | --- |
| Term | Coefﬁcients | Standard error | P-value ^b^ | F-value ^c^ |
| Intercept | 2612171 | 633184 | < 0.001 |  |
| Treat. _(Food restricted)_^a^ | 802354 | 688156 | 0.256 | 0.701 |
| Treat. _(Physically stressed)_^a^ | 608652 | 691503 | 0.388 | 0.701 |
| IBM | 32466 | 22543 | 0.163 | 2.075 |
| Response= lymphocytes | | | | |
| Intercept | 2681.58 | 672.87 | < 0.001 |  |
| Treat. _(Food restricted)_^a^ | 314.21 | 454.09 | 0.496 | 0.204 |
| Treat. _(Physically stressed)_^a^ | -37.00 | 465.87 | 0.937 | 0.204 |
| IBM | 27.82 | 32.96 | 0.408 | 0.712 |
| Response= neutrophils | | | | |
| Intercept | 2392.10 | 645.22 | 0.001 |  |
| Treat. _(Food restricted)_^a^ | -126.54 | 435.43 | 0.774 | 0.163 |
| Treat. _(Physically stressed)_^a^ | -283.61 | 446.73 | 0.532 | 0.163 |
| IBM | 25.32 | 31.61 | 0.432 | 0.642 |
| Response= monocytes | | | | |
| Intercept | 132.06 | 77.27 | 0.101 |  |
| Treat. _(Food restricted)_^a^ | 16.71 | 78.02 | 0.832 | 0.028 |
| Treat. _(Physically stressed)_^a^ | 22.059 | 78.55 | 0.781 | 0.028 |
| IBM | -0.624 | 3.01 | 0.838 | 0.068 |
| Response= neutrophil : lymphocyte ratio | | | | |
| Intercept | 132.06 | 77.27 | 0.101 |  |
| Treat. _(Food restricted)_^a^ | 16.71 | 78.02 | 0.832 | 0.812 |
| Treat. _(Physically stressed)_^a^ | 22.06 | 78.55 | 0.781 | 0.812 |
| IBM | -0.624 | 3.01 | 0.838 | 0.062 |
| Response= spleen mass | | | | |
| Intercept | 21.88 | 7.47 | 0.008 |  |
| Treat. _(Food restricted)_^a^ | -10.29 | 5.04 | 0.053 | 4.599 |
| Treat. _(Physically stressed)_^a^ | -7.36 | 5.17 | 0.169 | 4.599 |
| IBM | 2.06 | 0.36 | < 0.001 | 31.53 |
| Response= plasmatic proteins | | | | |
| Intercept | 3.23 | 0.51 | < 0.001 |  |
| Treat. _(Food restricted)_^a^ | 0.64 | 0.34 | 0.073 | 1.36 |
| Treat. _(Physically stressed)_^a^ | 0.37 | 0.35 | 0.309 | 1.36 |
| IBM | 0.08 | 0.02 | 0.003 | 11.55 |
| Response= albumin | | | | |
| Intercept | 2.918 | 0.32 | < 0.001 |  |
| Treat. _(Food restricted)_^a^ | 0.246 | 0.22 | 0.273 | 0.81 |
| Treat. _(Physically stressed)_^a^ | 0.233 | 0.22 | 0.309 | 0.81 |
| IBM | -0.000 | 0.01 | 0.989 | 0.00 |
| Response= globulins | | | | |
| Intercept | 0.311 | 0.32 | 0.511 |  |
| Treat. _(Food restricted)_^a^ | 0.398 | 0.22 | 0.219 | 0.42 |
| Treat. _(Physically stressed)_^a^ | 0.132 | 0.22 | 0.686 | 0.42 |
| IBM | 0.085 | 0.01 | 0.001 | 13.7 |
| Response= albumin:globulin ratio | | | | |
| Intercept | 2.630 | 0.43 | < 0.001 |  |
| Treat. _(Food restricted)_^a^ | -0.098 | 0.34 | 0.775 | 0.001 |
| Treat. _(Physically stressed)_^a^ | 0.099 | 0.34 | 0.775 | 0.001 |
| IBM | -0.050 | 0.02 | 0.019 | 6.34 |

^a^ Simple contrasts – reference level: control (the coefficients reﬂect comparison with control groups).

^b^ P-values obtained from Markov chain Monte Carlo samples (*pvals.fnc* function in R).

^c^ For the factor ‘Treatment’: numerator degrees of freedom = 2; denominator degrees of freedom = 24.
