# Supplementary material for: Parasitism and Physiological Trade-Offs in Stressed Capybaras
Source: PLoS One. 2013 Jul 24;8(7):e70382. doi: 10.1371/journal.pone.0070382 (PMC3722164; doi:10.1371/journal.pone.0070382)
Supplement: Table S3 — Linear models showing the relationship between parasite load and body condition. The inclusion of the interaction term Treatment*Parasite allowed assessing whether there was a change in the tolerance to parasites induced by stress. Body condition was negatively associated with Trichuris sp. and Strongyloides chapini. In no case, the interaction term Treatment*Parasite was significant, indicating no influence of treatments on parasite tolerance. (DOC) [file pone.0070382.s004.doc]

Table S3. **Linear models showing the relationship between parasite load and body condition.** The inclusion of the interaction term Treatment*Parasite allowed assessing whether there was a change in the tolerance to parasites induced by stress. Body condition was negatively associated with *Trichuris* sp and *Strongyloides chapini*. In no case, the interaction term Treatment*Parasite was significant, indicating no influence of treatments on parasite tolerance.

| Model = Bodycondition ~ Treatment* *S. chapini*. | | | |
| --- | --- | --- | --- |
| Term | Coefﬁcients | Standard error | P-value |
| Intercept | 7.591 | 0.353 | < 0.001 |
| Treat. _(Food restricted)_^a^ | -2.275 | 0.471 | < 0.001 |
| Treat. _(Manipulated)_^a^ | -0.515 | 0.488 | 0.303 |
| *S. chapini* | -0.002 | 0.001 | 0.045 |
| *S. chapini*: Treat. _(Food restricted)_ | 0.001 | 0.001 | 0.355 |
| *S. chapini*: Treat _(Manipulated)_ | -0.003 | 0.003 | 0.385 |
| Model = Bodycondition ~ Treatment* *E. hydrochaeris* | | | |
| Intercept | 7.654 | 0.595 | < 0.001 |
| Treat. _(Food restricted)_^a^ | -2.495 | 0.810 | 0.006 |
| Treat. _(Manipulated)_^a^ | -1.079 | 0.850 | 0.219 |
| *E. hydrochaeris* | -0.011 | 0.009 | 0.395 |
| *E. hydrochaeris* : Treat. _(Food restricted)_ | 0.008 | 0.015 | 0.575 |
| *E. hydrochaeris* : Treat _(Manipulated)_ | 0.016 | 0.019 | 0.287 |
| Model = Bodycondition ~ Treatment **Trichostrongylus* sp. | | | |
| Intercept | 7.355 | 0.337 | <0.001 |
| Treat. _(Food restricted)_^a^ | -2.152 | 0.467 | <0.001 |
| Treat. _(Manipulated)_^a^ | -0.433 | 0.497 | 0.394 |
| *Trichostrongylus* sp. | -0.010 | 0.006 | 0.120 |
| *Trichostrongylus* sp. : Treat. _(Food restricted)_ | -0.009 | 0.016 | 0.597 |
| *Trichostrongylus* sp. : Treat _(Manipulated)_ | -0.088 | 0.100 | 0.391 |
| Model = Bodycondition ~ Treatment *Viannaidae | | | |
| Intercept | 7.453 | 0.402 | <0.001 |
| Treat. _(Food restricted)_^a^ | -2.112 | 0.537 | <0.001 |
| Treat. _(Manipulated)_^a^ | -1.229 | 0.668 | 0.080 |
| Viannaidae | -0.007 | 0.005 | 0.217 |
| Viannaidae : Treat. _(Food restricted)_ | -0.010 | 0.013 | 0.427 |
| Viannaidae : Treat _(Manipulated)_ | 0.059 | 0.043 | 0.181 |
| Model = Bodycondition ~ Treatment* *Trichuris sp*. | | | |
| Intercept | 7.441 | 0.28967 | < 0.001 |
| Treat. _(Food restricted)_^a^ | -2.013 | 0.40672 | < 0.001 |
| Treat. _(Manipulated)_^a^ | -0.513 | 0.40672 | 0.222 |
| *Trichuris sp.* | -0.135 | 0.05859 | 0.032 |
| *Trichuris sp.*: Treat. _(Food restricted)_ | -0.201 | 0.13456 | 0.151 |
| *Trichuris sp.*: Treat _(Manipulated)_ | -0.103 | 0.14679 | 0.491 |

^a^Simple contrasts – reference level: control (the coefficients reﬂect comparison with control groups).
